# Genetic Diversity of Norovirus and Sapovirus Outbreaks in Long-Term Care Facilities in Quebec, Canada, 2011–2016

**DOI:** 10.3390/v18010085

**Published:** 2026-01-08

**Authors:** Émilie Larocque, Yvan L’Homme, Hugues Charest, Christine Martineau

**Affiliations:** 1 Food Virology National Reference Centre, St. Hyacinthe Laboratory, Canadian Food Inspection Agency, St. Hyacinthe, QC J2S 8E3, Canada; 2 Laboratoire de santé publique du Québec (LSPQ), Institut National de Santé Publique du Québec, Montréal, QC H9X 3R5, Canadachristine.martineau@nrcan-rncan.gc.ca (C.M.); 3 Department of Microbiology, Infectiology and Immunology, Faculty of Medicine, Montréal University, Montréal, QC H3T 1J4, Canada

**Keywords:** norovirus, sapovirus, genetic diversity, genotype, molecular surveillance, acute gastroenteritis (AGE), outbreak

## Abstract

Norovirus (NoV) and sapovirus (SaV) are major viral pathogens causing acute gastroenteritis (AGE) in both children and adults in developed countries and are also responsible for large-scale outbreaks. However, in Quebec, Canada, there are limited and updated data with respect to the genotypes circulating and implicated in outbreaks, particularly for SaV. This study aimed to investigate the genetic diversity and genotype predominance of NoVs and SaVs associated with AGE outbreaks in Quebec, Canada. Confirmed NoV and SaV outbreaks from long-term care facilities and hospital settings between September 2011 and April 2016 were investigated (*n* = 252). NoVs and SaVs were genetically diverse: 21 RdRp-capsid combinations were identified, of which 10 are recombinants. NoV GII.4 New Orleans[P4 NewOrleans] was the predominant genotype from 2011 to 2013, and GII.4 Sydney[P31] was the predominant genotype from 2013 to 2015. In 2015–2016, no single genotype predominated; instead, GII.17[P17], GII.4 Sydney[P16], GII.4 Sydney[P31], and SaV GI.2 strains were co-circulating at similar frequencies. Notably, emerging global genotypes including GII.17[P17], GII.4 Sydney[P16], GII.2[P16], and GII.4 San Francisco[P31] were detected for the first time in Quebec. These findings may contribute to an enhanced understanding of NoV and SaV infection and spread, and to the development of candidate vaccines.

## 1. Introduction

Noroviruses (NoVs) and Sapoviruses (SaVs) are important etiological agents of acute gastroenteritis (AGE) which spread by contact with infected individuals, contaminated food, or fomites. It is estimated that 684 million people become sick every year from NoV infections: it is the most common cause of AGE across all age groups in both outbreaks and sporadic cases around the world [1]. The elderly are particularly vulnerable to NoV infection as it is associated with increased hospitalization and mortality rates [2,3]. SaV infections are less common than those attributed to NoVs and are most often associated with AGE in young children and adults >65 years of age [4,5]. There is currently no licenced vaccine available for the prevention of NoV or SaV infection or illness, but several candidates are in clinical and preclinical development [6].

NoVs and SaVs are non-enveloped viruses belonging to the family *Caliciviridae*. Both viruses possess a positive-sense RNA genome of approximately 7.5 kb in length, of which the 5′ end is covalently linked to a viral protein, VPg, and the 3′ end is polyadenylated. Nevertheless, their genomic organization differs slightly. The NoV genome consists of three overlapping open reading frames (ORFs) with ORF-1 encoding six non-structural proteins including the RNA-dependent RNA polymerase ([RdRp]), NS7 region). ORF-2 encodes the major capsid protein, VP1, and ORF-3 encodes the minor capsid protein, VP2. Conversely, the SaV genome usually consists of two ORFs. The *polyprotein* gene from ORF1 contains the coding regions for non-structural proteins such as RdRp and the VP1 capsid. ORF2 is thought to encode the minor structural protein, VP2 [7].

Complete VP1 amino acid sequences and RdRp nucleotide sequences are used for classification of noroviruses, based on a 2× standard deviation (sd) criteria, into genogroups as well as P (polymerase)-groups and further into genotypes and P-types [8,9]. Of the 10 currently recognized genogroups (GI-GX), GI, GII, GIV, GVIII, and GIX infect humans, encompassing over 30 genotypes [9]. The majority of NoV gastroenteric illness outbreaks over the last two decades have been attributed to GII.4 strains, including GII.4 DenHaag[P4 DenHaag], GII.4 New Orleans[P4 New Orleans], GII.4 Sydney[P31] (previously Pe), and GII.4 Sydney[P16] [10,11,12]. In contrast, the proportion of outbreaks associated with non-GII.4 genotypes has increased in recent years [13,14]: a wave of GII.17 norovirus outbreaks and sporadic infections of acute gastroenteritis was reported in many European countries and the United States during the 2023/24 season [15,16].

Reminiscent of NoVs, SaVs are also highly diverse [17] and classified into 19 genogroups (GI–GXX) based on complete VP1 nucleotide sequences [18,19]. Human SaVs are limited to GI, GII, GIV, and GV, each of which are further subdivided into 18 genotypes [20,21]. SaV-GI and -GII are the most two common human genogroups in low- and middle-income countries [22,23]. RdRp-VP1 recombinants have been identified, albeit less frequently than in NoVs [24]. A dual-typing system for SaVs has also been proposed but is not yet a standard practice [21,23].

Currently, genomic data on NoV and SaV genotypes circulating in Canada are sparse and focused on the western part of the country [4,25,26]. Molecular surveillance of circulating strains is crucial to understanding transmission patterns, burden of infections, population immunity, as well as the development of candidate vaccines and antivirals. Herein, we report the prevalence, genetic diversity, emerging strains, and recombination of NoV and SaV in AGE outbreaks in long-term care facilities and hospital settings in Quebec, Canada, between 2011 and 2016.

## 2. Materials and Methods

### 2.1. Stool Specimens

An investigation is launched by the Laboratoire de santé publique du Québec when an institution reports ≥2 cases of AGE to public health officials. When a viral agent is suspected, total RNA is extracted from 200 µL of 20% stool suspension using the NucliSENS EasyMAG™ (Biomérieux, Ville Saint-Laurent, QC, Canada) following the manufacturer’s recommendations and tested for NoV GI, NoV GII and SaV by real-time RT-PCR assays according to previously published methods [27,28]. RNA extracts from NoV GI-, NoV GII- or Sav-positive samples are kept at the Laboratoire de santé publique du Québec at −80 °C until further analysis. Samples representative of 252 documented NoV GI-, NoV GII-, or Sav-positive AGE outbreaks that occurred in the province of Quebec in long-term care facilities and hospital settings from 2011 to 2016 were selected for genetic analyses. Yearly data was analyzed from September 1 to August 31. Sample collection in 2016 ended in May.

### 2.2. Dual Typing RT-PCR

Genotyping was conducted by the Canadian Food Inspection Agency. One positive RNA extract was selected by random draw among positive samples and subjected to genotyping unless multiple viral agents were detected by RT-qPCR. In such instances, all available samples from the outbreak (ranging from 2 to 7) were subjected to genotyping (Table A1). If the same genotype was identified in multiple samples within a single outbreak, it was reported only once for that outbreak. The samples were amplified by two-step RT-PCR using one of three sets of primers (Table 1) targeting the RdRp region of ORF1, and the complete VP1 region of ORF2. Primers were selected, modified, or newly designed based on in silico binding analyses using the reference sequences provided in Supplementary Material Table S1. Two microliters of RNA was reverse transcribed into cDNA using an oligo(dT)20 primer and SuperScript™ IV reverse transcriptase (Thermo Fisher Scientific, Asheville, NC, USA) in a 20 µL reaction volume according to the manufacturer’s instructions. After treatment with 5 U of RNase H (Thermo Fisher Scientific, Asheville, NC, USA) for 20 min at 37 °C, amplification by PCR was performed using Platinum™ Taq DNA polymerase (Thermo Fisher Scientific, Asheville, NC, USA). Reactions were carried out in a final 50 µL volume containing 1× Platinum™ Taq Buffer, 0.2 µM of forward and reverse primers (listed in Table 1), and 4 µL of cDNA. PCR cycling parameters for NoV GI and NoV GII involved an initial denaturation step of 2 min at 94 °C, and 40 cycles of 30 s at 94 °C, 55 s at 50 or 49 °C, 2 min 30 s at 72 °C, and a final extension of 10 min at 72 °C, respectively. PCR cycling parameters for SaV involved an initial denaturation step of 3 min at 94 °C, and 40 cycles of 30 s at 94 °C, 30 s at 54 °C, 3 min at 72 °C, and a final extension of 10 min at 72 °C. PCR products were analyzed by gel electrophoresis on a 1% agarose gel (Thermo Fisher Scientific, Asheville, NC, USA). Positive PCR reactions were purified using a QIAquick PCR purification kit (QIAGEN, Toronto, ON, Canada) according to instructions. The expected amplicon sizes were ~2.4 kb for NoV GI and NoV GII, and ~2.5 kb for SaV.

### 2.3. Sequencing

The purified PCR products were subjected to sequencing on the Illumina MiSeq instrument (Illumina, Victoria, BC, Canada). Libraries were prepared using the Nextera XT Library Preparation Kit (Illumina, Victoria, BC, Canada) according to the manufacturer’s instructions. Paired-end dual-indexed sequencing runs of 2 × 75 cycles were conducted on pooled libraries with 1% PhiX (Illumina Victoria, BC, Canada). Raw sequence reads were processed in CLC Genomics Workbench v10.0.1 (QIAGEN, Toronto, ON, Canada). First, leftover adapter sequences were removed from reads and trimmed at a quality limit of 0.001 (Q > 30) and subsequently for ambiguity. Reads were sampled (35,000 reads/sample) to obtain an overall coverage of 1000×. The resulting paired-reads were assembled de novo using an automatic bubble (50) and word (20) sizes and a minimum contig length of 200 nt after scaffolding. Contigs with an overall depth threshold of <100× were excluded from the analysis to ensure certainty of results. Consensus sequences were extracted from contigs: regions of low coverage (≤9×) were removed, and ambiguity codes were inserted to resolve conflicts above the noise threshold (0.1). Internal primer sequences listed in Table 1 were also removed. Consensus sequences obtained from outbreak samples were deposited in GenBank under the accession numbers PX720646-PX720657, PX732916-PX733095, and PX737900-PX737926.

### 2.4. Genotyping

Norovirus genotypes were determined by dual-typing the polymerase region (P-type) and the capsid (genotype) using the automated online norovirus genotyping tool accessed on 15–16 December 2021 (http://www.rivm.nl/mpf/norovirus/typingtool) [31]. Sapovirus sequences were genotyped based on a 420 nt typing region encoding VP1 using the Human Calicivirus Typing (HuCaT) tool accessed on 17 December 2021 (https://calicivirustypingtool.cdc.gov/) [32].

### 2.5. Recombination

A strain was defined as a recombinant when the ORF-1 and ORF-2 nucleotide sequences clustered into separate phylogenetic groups. Recombinant sequences were then aligned with closely related GenBank reference sequences with MUSCLE using MEGA [33]. Recombination breakpoints in the parental sequences were identified with the software Recombination Detection Program v4.96 (RDP4) [34] using default settings including primary screening using the RDP, GENECONV, MaxChi, Chimera, and 3Seq methods, and secondary screening using the BootScan and SiScan methods. A general recombination detection *p*-value threshold was set to 0.05.

### 2.6. Phylogenetic Analysis

Phylogenetic trees of the RdRp and VP1 regions were built separately using the sequences from this study, and reference sequences obtained from the GenBank database. First, sequence alignments were constructed using MUSCLE (MUltiple Sequence Comparison by Log-Expectation) as a plug-in in CLC Genomics Workbench. All aligned sequences were trimmed for the partial RdRp and complete VP1. Phylogenetic analyses were conducted using the neighbour-joining (NJ) method implemented in CLC Genomics Workbench with the most appropriate nucleotide substitution model based on the Model Testing tool. Bootstrapping values at the nodes were obtained by performing 1000 replicate analyses. The percent identity among nucleotide sequences was compared using CLC Genomics Workbench.

## 3. Results

From September 2011 to May 2016, real-time RT-PCR results showed that most of the 252 documented viral AGE outbreaks were caused by NoV GII (202/252 or 80.2%), followed by SaV (24/252 or 9.5%), and NoV GI (15/252 or 6.0%), while some were mixed NoV GII/SaV (7/252 or 2.8%) or NoV GI/GII (4/252 or 1.6%). Mixed outbreaks were only detected in 2014–2015 and 2015–2016.

**Figure 1:**
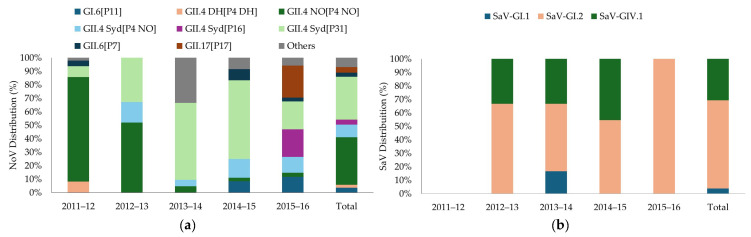
Yearly distribution of NoV (**a**) and SaV (**b**) genotypes from AGE outbreaks in Quebec, Canada, 2011–2016. Different NoV and SaV genotypes are represented by different colours. Genotypes with overall frequencies ≤ 1% were grouped under “others”. Syd, Sydney 2012; NO, New Orleans 2009; DH, Den Haag 2006.

The genotypes based on dual typing of the partial RdRp and complete VP1 gene regions are shown in Figure 1 and Table 2. A total of 218 positive outbreak samples were genotyped successfully. Most outbreak samples that could not be genotyped failed due to amplification failure. Additionally, four NoV GII outbreak samples were excluded because of technical errors and one NoV GI outbreak sample was not genotyped due to insufficient coverage based on exclusion criteria. The predominant genotype overall was NoV GII.4 New Orleans[P4NewOrleans] followed by GII.4 Sydney[P31], accounting for 31.2% (68/218) and 38.0% (61/218) of genotyped sequences, respectively.

In addition to the prevalent genotypes, diverse genotypes were identified throughout the study period, including the detection of 6 NoV GI, 12 NoV GII, and 3 SaVs. Temporal trends were also observed. From 2011 to 2013, NoV GII.4 New Orleans[P4NewOrleans] was the single predominant genotype, followed by GII.4 Sydney[P31]. Its prevalence suddenly dropped during 2013–2014. Meanwhile, GII.4 Sydney[P31], first detected in 2011–2012, increased in prevalence and became the predominant genotype from 2013 to 2015 followed by SaV-GI.2 in 2014–2015. In 2015–2016, no single predominant genotype was observed, but four genotypes were circulating at similar frequencies: GII.17[P17], GII.4 Sydney[P16], GII.4 Sydney[P31]. and SaV-GI.2. Notably, other genotypes detected during the study period included GII.4 Den Haag 2006b, which caused outbreaks from 2011 to 2013 (*n* = 4) before disappearing the following year, and GII.4 Sydney[P4NewOrleans], which was consistently detected throughout the study period (*n* = 18). GII.6[P7] was detected in 2011–2012 and then again in 2014–2015 and 2015–2016 (*n* = 6), while GI.6[P11] emerged during 2014–2015 (*n* = 7). Additionally, SaV-GIV.1 was steadily detected during the study period but disappeared in 2015–2016 (*n* = 8). NoV GI.2[P2], GI.3[P3], GI.5[P5], GI.9[P9], GII.1[P33], GII.8[P8], GII.13[P16], and SaV-GI.1 were also detected, albeit at frequencies below 1%, with distribution levels varying according to the year. From 2011 to 2013, little NoV diversity was observed, with 3–5 genotypes in circulation and increasing to 8–9 genotypes in circulation from 2013 to 2014 onward.

Dual typing of the RdRp and VP1 gene regions using the web-based norovirus typing tool revealed that 55.5% (106/191) of NoV sequences represented recombinant genotypes. This includes genotypes GI.3[P10], GI.6[P11], GII.1[P33], GII.2[P16], GII.4 Sydney[P4NewOrleans], GII.4 Sydney[P16], GII.4 Sydney[P31], GII.4 San Francisco[P31], GII.6[P7], and GII.13[P16]. Recombination events were confirmed using the RDP4 program and were found at or near the ORF1/ORF2 junction when compared to parental strains (Table A2). Breakpoints could not be found in two GII.6[P7] sequences (GE-61027, GE-61417). Due to the unavailability of nonrecombinant parental sequences for GI.3[P10], GI.6[P11] and GII.4 San Francisco [P31] in the database, further investigation of recombination events was not possible.

**Figure 2:**
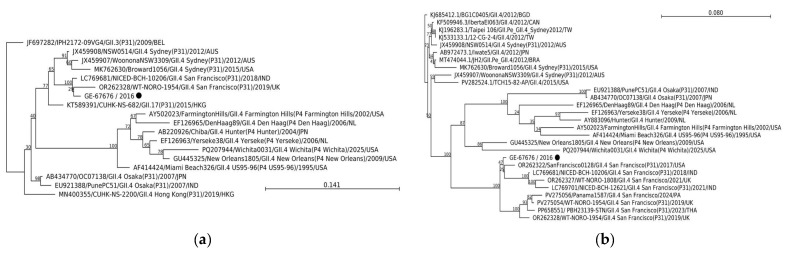
Phylogenetic trees of NoV GII.4 San Francisco[P31] RdRp (**a**) and VP1 (**b**) gene regions. The trees were constructed by the neighbour-joining method with the K80 + G + T (RdRP) and GTR + G + T (VP1) parameter model nucleotide substitution model, respectively, with 1000 bootstrap replicates. Bootstrap values are shown at the branch nodes. All reference sequence names are formatted as GenBank accession number/sample name/genotype(strain)/year/country (JPN, Japan; CHN, China; USA, United States; KOR, Korea; AUS, Australia; RUS, Russia; CAN, Canada; BEL, Belgium; HKG, Hong Kong; KHM, Cambodia; IND, India; NL, Netherlands; BGD, Bangladesh; TW, Taiwan; BRA, Brazil; UK, United Kingdom; THA, Thailand; PA, Panama). Strains obtained from our study are labelled as sample name/year with a black circle.

Notably, four emerging global NoV strains were detected during the last annual period of the study: GII.17[P17], GII.4 Sydney[P16], GII.2[P16], and GII.4 San Francisco[P31]. Phylogenetic trees were constructed with the RdRP and VP1 gene regions of epidemiological relevant GII.4 San Francisco [P31] and GII.17[P17] strains from our study with those previously reported worldwide to determine their relationship. The sole GII.4 San Francisco[P31] NoV (GE-67676/2016) was clustered in both gene regions, with the novel GII.4 San Francisco[P31] strain detected in Indonesia, the United Kingdom, and the United States (Figure 2). The VP1 shared 98.95% nt identity with the GII.4 San Francisco[P31] reference strain isolated from the USA in 2017 (OR262322).

**Figure 3:**
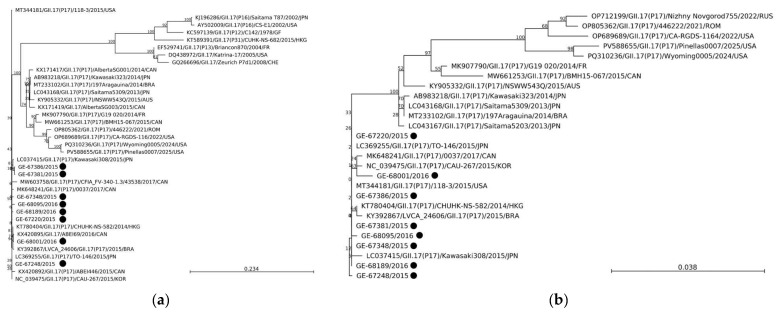
Phylogenetic trees of NoV GII.17[P17] RdRp (**a**) and VP1 (**b**) gene regions. The trees were constructed by the neighbour-joining method with the GTR + G + T parameter model nucleotide substitution model, respectively, with 1000 bootstrap replicates. Bootstrap values are shown at the branch nodes. All reference sequence names are formatted as GenBank accession number/sample name/genotype(strain)/year/country (JPN, Japan; Hong Kong, HKG; CHE, Switzerland; USA, United States; KOR, Korea; AUS, Australia; CAN, Canada; BRA, Brazil; FR, France; ROM, Romania; GF, French Guiana; RUS, Russia). Strains obtained from our study are labelled as sample name/year with a black circle.

The 8 GII.17[P17] NoV from this study (GE-67220, GE-67248, GE-67348, GE-67381, GE-67396, GE-68001, GE-68095, GE-68189) showed a mean nucleotide identity of 99.54% (99.3–99.9%). Phylogenetic analysis of the partial RdRp and full-length VP1 nucleotide sequences indicated that all the reported NoV with GII.17[P17] sequences were clustered with the Kawasaki 308 cluster (>99% nt identity with LC037415 reference strain) (Figure 3).

**Figure 4:**
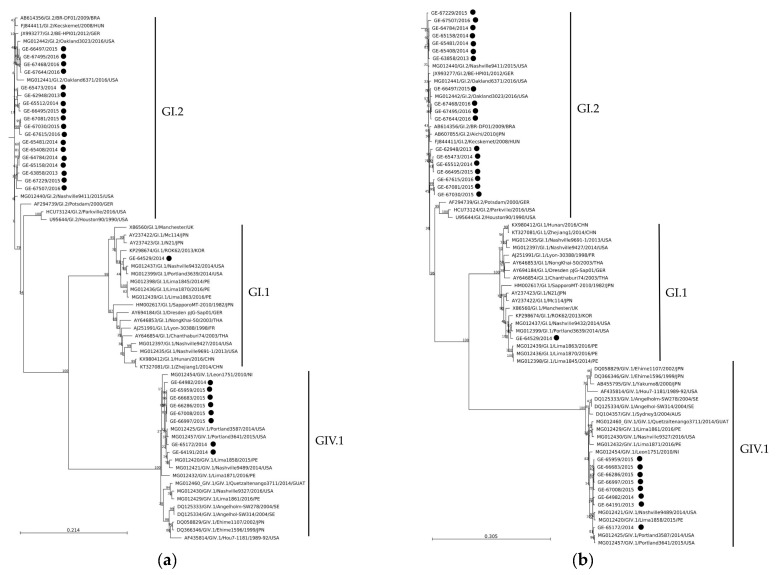
Phylogenetic trees of SaV RdRp (**a**) and VP1 (**b**) gene regions. The trees were constructed by the neighbour-joining method with the K80 + G + T (RdRP) and GTR + G +T (VP1) parameter model nucleotide substitution model, respectively, with 1000 bootstrap replicates. Bootstrap values are shown at the branch nodes. All reference sequence names are formatted as GenBank accession number/sample name/genotype(strain)/year/country (BRA, Brazil; HUN, Hungary; GER, Garmany; UK, United Kingdom; PE, Peru; FR, France; NI, Nicaragua; GUAT, Guatemala; JPN, Japan; CHN, China; USA, United States; THA, Thailand; KOR, Korea; RUS, Russia; CAN, Canada; SE, Sweden; AUS, Australia). Strains obtained from our study are labelled as sample name/year with a black circle.

A total of 27 sapoviruses were identified in this study: 1 SaV-GI.1, 18 SaV-GI.2 and 8 SaV-GIV.1. Comparisons of the 2.5 kb-long nucleotide sequences showed that the 18 SaV-GI.2 sapoviruses shared 97.9% nucleotide identity (96.5–100%). As depicted in Figure 4, the phylogenetic trees inferred from partial RdRp and full-length VP1 sequences showed that all the present SaV sequences separated into clusters with the expected genotype in both gene regions identified by the online automatic typing tool (Figure 4a,b). The 18 SaV-GI.2 sequences formed one big cluster with SaV-GI.2 reference strains from multiple countries, including the United States, Hungary, Germany, Japan, and Brazil. The single SaV-GI.1 sequence from this study was clustered with two SaV-GI.1 reference strains from the United States. The SaV-GIV.1 sequences from this study shared 99.15% (98.02–99.96%) nucleotide identity and formed a distinct cluster with strains from the United States and Peru in the VP1 gene.

## 4. Discussion

NoV and SaV are a common cause of AGE in children and adults in developed countries. They are also responsible for major outbreaks of gastroenteritis in closed settings, especially in facilities caring for the elderly [4]. In Quebec, Canada, outbreaks of infectious viral AGE are investigated by the Laboratoire de santé publique du Québec, but detailed information on genotypes circulating in the province is lacking. This study fills this knowledge gap by providing information of both polymerase and capsid genes for NoVs and SaVs circulating in Quebec, Canada, from 2011 to 2016, through a five-year continuous surveillance of AGE outbreaks in long-term care facilities and hospital settings.

The findings from this study revealed significant shifts in the distribution of circulating NoV and SaV genotypes during outbreaks. The replacement of NoV GII.4 New Orleans[P4 NewOrleans] as the dominant genotype by GII.4 Sydney[P31] reflects the previously described temporal trends of GII.4 noroviruses, although this shift occurred in 2013–2014, happening two seasons later than the one observed in the United States [16]. Another notable shift occurred in 2015–2016 and is characterized by the absence of a single predominant genotype. The reason for the lack of a sole predominant strain is unclear, but it is tempting to speculate that it has led to the takeover by the GII.4 Sydney[P16] genotype in subsequent years. This novel recombinant emerged in late 2014 and was described around the world, including Canada (Province of Alberta), the United States, Germany, Italy, Japan, and South Korea [12,35,36,37,38,39]. By 2016, GII.4 Sydney[P16] became the predominant genotype in many western countries [12,35,38,40,41,42].

High genotypic diversity of NoV strains has previously been reported in outbreaks settings which occurred in long-term care facilities in Canada and other countries [43,44,45]. Here, the number of annual genotypes in circulation doubled in the last three years of this study. Moreover, the majority of NoVs detected were recombinant strains which is in agreement with other studies showing that most AGE outbreaks in recent years are attributed to recombinant NoV strains, with recombination occurring between ORF1 and ORF2 [46,47]. However, dual typing was not frequently performed prior to 2010, making this comparison challenging. All recombinants detected had been reported previously from patients with acute gastroenteritis [35,46,48,49,50,51].

A novel GII.4, GII.4 San Francisco, has been identified recently in the United States, Gabon, South Africa, the United Kingdom, and Indonesia, covering the period from 2017 to 2020 [51]. Here, we detected GII.4 San Francisco in February 2016 circulating as a recombinant strain with a P31-type RdRp. To our knowledge, this is the first report of GII.4 San Francisco in Canada and it further supports its global distribution. This novel GII.4 capsid shows mutations on major antigenic sites and a single amino acid insertion next to the antigenic site A [51]. It presents distinct antigenic profiles, and in terms of HBGA binding profiles, bound to porcine gastric mucin III and human saliva [52]. Since 2017, a total of three novel GII.4 clusters emerged based on amino acids changes in VP1: GII.4 San Francisco[P31], GII.4 Allegany[P31], and GII.4 Wichita[P4] [16]. Continued molecular surveillance of GII.4 is required to assess the burden and spread of these most recent variants.

In 2014–2015, a novel GII.17 norovirus variant, Kawasaki 308 (GII.17[P17]), emerged in Asia and spread globally [37,53,54,55]. It even completely replaced GII.4 Sydney 2012 in several countries in Asia in 2014 [53]. This variant is considered an immune-escape variant with a faster mutation rate than GII.4 [56]. Here, GII.17 Kawasaki 308 was first detected in Quebec in early winter 2016, causing a total of eight outbreaks in long-term care facilities that year. Thus, since its emergence, the GII.17[P17] Kawasaki 308 has caused a number of outbreaks across Canada [35,57]. During 2023–2024, an increase in GII.17 cases and outbreaks was reported in many countries, including the United States [15]. During the 2024–2025 season, 75% of the outbreaks uploaded to CaliciNet were GII.17 replacing, for the first time, GII.4 (including GII.4 Sydney, GII.4 San Francisco, and GII.4 Wichita grouped together) as the predominant norovirus outbreak strain in the United States [16]. However, phylogenetic analysis revealed most of the GII.17 from 2023 to 2024 clustered closely with the GII.17 Romania-2021 variant in both VP1 and RdRp regions [58]. Continued monitoring of GII.17 in Quebec, Canada, is essential to track the local spread and evolution of GII.17.

As for SaV, the findings from this study indicate that SaV-GI.2 was the dominant SaV genotype. Previous studies have also identified SaV-GI.2 as the most prevalent genotype in sporadic and outbreak-related SaV gastroenteritis [59,60,61,62,63,64]. There were also reports of the SaV-GI.2 genotype causing infection in the United States [45], China [65], Taiwan [66], Japan [67], and Germany [68]. In contrast, Zhuo et al. reported SaV-GI.1 as the predominant genotype in pediatric sporadic case of SaV-associated AGE in Canada (Alberta) between 2014 and 2018 (36%), followed by SaV-GI.2 (18%), SaV-GII.5 (8%), and SaV-GII.3 (6%) [25]. These findings differ from ours, where SaV GI.1 was detected only once in 2013–2014, suggesting temporal and/or geographical variations in SaV genotype patterns. Interestingly, Zhuo et al. also reported that SaV-GI.2 overtook GI.1 as the predominant strain in 2016–2017, with high detection rates persisting into April. SaV-GI.2 was also a major cause of AGE outbreaks, in Quebec between 2015 and 2016. Rare genotypes, including SaV-GII.1, SaV-GII.2, SaV-GV.1, SaV-GII.4, SaV-GIV.1, SaV-GI.3, and SaV-GI.7 were also identified in pediatric sporadic case of SaV-associated AGE between 2014 and 2018 [25]. In this study, SaV-GIV.1 was the second most common SaV genotype, accounting for nearly one third of the SaVs, but was not detected in 2015–2016. Historically, this genotype was associated with increase outbreak activity in long-term care facilities and senior lodges in Canada (2004–2007) and the United States (2002–2009) [26,45]. Overall, these results highlight differences in SaV prevalence and indicate geographical and/or temporal changes in SaV genotype distribution. Although SaV recombinant strains have been reported [23], phylogenetic analysis of partial RdRp and full-length VP1 sequences showed all SaV sequences from this study clusters with their expected genotype in both regions, as identified by the online typing tool, suggesting nonrecombinant circulation.

Although extensive, the genetic diversity of the NoVs and SaVs reported in this study is most likely underestimated. For instance, noroviruses bearing a GII.3 capsid (GII.3[P12], GII.3[P21], GII.3[P16], which are usually highly represented worldwide including in North America [69], were not detected in this study. RT-PCR amplification biases cannot be completely ruled out, and suboptimal PCR primers are the most probable cause for the lack of certain genotypes in our study. Also, stool specimens are from outbreaks that occurred in long-term care facilities and hospital setting and genetic diversity of circulating strains might be less well represented in comparison to sporadic samples.

## 5. Conclusions

In summary, five years of molecular surveillance of NoV- and SaV-associated AGE outbreaks showed that genetic diversity of circulating NoVs and SaVs in Quebec, Canada, is extensive and genotype distribution trends vary according to the year. Detection of emerging global genotypes suggests frequent and rapid spread in the province. Ultimately, these findings may contribute to enhanced understanding of norovirus and sapovirus infection, leading to greater insight into transmission patterns, population immunity, burden of disease, and supporting vaccine design.

## Figures and Tables

**Table 1:** Primers used for genotyping NoV and SaV.

Target	Primer Name	Sequence 5′ to 3′	Position	Reference
NoV GI-II	p290IUB	GATTACTCCARGTGGGAYTCMAC	4568 ^a^	[29]
NoV GI	NoVGI-R3	CTCCAAWDATDGCTTGRGCCAT	6971 ^a^	This study
NoV GII	mGV132	GCDAHRAAAGCTCCNGCCATTA	6723 ^b^	This study
SaV	p290	GATTACTCCAAGTGGGACTCCAC	4354 ^c^	[30]
SaV	Sav1R	AATGAGTTGGTTNGTTGG	6868 ^c^	This study

^a–c^ Position of the 5′ end of the primer in the nucleotide sequence of Norwalk strain (M87661) [^a^], Lordsdale strain (X86557) [^b^], and Manchester strain (X86560) [^c^].

**Table 2:** Yearly distribution of genotypes of NoV- and SaV-associated outbreaks in Quebec, Canada, August 2011–May 2016.

Virus	Capsid Type	P-Type	2011–12	2012–13	2013–14	2014–15	2015–16	Total
NoV	GI.2	GI.2	0	0	2	0	0	2
	GI.3	GI.P3	0	0	0	1	0	1
	GI.3	GI.P10	0	0	1	0	0	1
	GI.5	GI.P5	0	0	0	1	0	1
	GI.6	GI.P11	0	0	0	3	4	7
	GI.9	GI.P9	0	0	1	0	0	1
	GII.1	GII.P33 (GII.Pg)	1	0	0	1	0	2
	GII.2	GII.P16	0	0	0	0	1	1
	GII.4 DH	GII.P4 DH	4	0	0	0	0	4
	GII.4 NO	GII.P4 NO	38	27	1	1	1	68
	GII.4 Syd	GII.P4 NO	0	8	1	5	4	18
	GII.4 Syd	GII.P16	0	0	0	0	7	7
	GII.4 Syd	GII.P31	4	17	12	21	7	61
	GII.4 SF	GII.P31	0	0	0	0	1	1
	GII.6	GII.P7	2	0	0	3	1	6
	GII.8	GII.P8	0	0	1	0	0	1
	GII.13	GII.P16	0	0	2	0	0	2
	GII.17	GII.P17	0	0	0	0	8	8
SaV	GI.1	-	-	0	1	0	0	1
	GI.2	-	-	2	3	7	6	18
	GIV.1	-	-	1	2	5	0	8
		Total	49	55	27	47	40	218

SF, San Francisco; Syd, Sydney 2012; NO, New Orleans 2009; DH, Den Haag 2006.

## Data Availability

The original data presented in the study will be openly available in GenBank under the accession numbers PX720646-PX720657, PX732916-PX733095, and PX737900-PX737926.

## Supplementary Materials

The following supporting information can be downloaded at https://www.mdpi.com/article/10.3390/v18010085/s1, Table S1: NCBI Reference Genomes Used for NoV and SaV Primer Design.

## Abbreviations

The following abbreviations are used in this manuscript:

AGE	Acute Gastroenteritis
SaV	Sapovirus
NoV	Norovirus
RNA	Ribonucleic acid
cDNA	Complementary strand deoxyribonucleic acid
ORF	Open Reading Frame
RdRp	RNA dependant RNA polymerase
RT-PCR	Reverse transcription polymerase chain reaction
nt	Nucleotide

## Appendix A

**Table A1:** Number of norovirus- and sapovirus-positive GE outbreaks and samples successfully genotyped between 2011 and 2016.

Year	NoV GI Outbreaks	NoV GI Samples	NoV GII Outbreaks	NoV GII Samples	SaV Outbreaks	SaV Samples	Mixed GI/GII Outbreaks	Mixed GI/GII Samples	Mixed GII/SaV Outbreaks	Mixed GII/SaV Samples	Total Outbreaks	Total Samples
2011–12	0/0	0/0	49/61	49/61	0/0	0/0	0/0	0/0	0/0	0/0	49/61	49/61
2012–13	0/4	0/4	52/67	52/67	3/4	3/4	0/0	0/0	0/0	0/0	55/75	55/75
2013–14	4/6	4/6	17/25	17/25	6/9	6/9	0/0	0/0	0/0	0/0	27/40	27/40
2014–15	2/2	2/2	23/26	23/26	7/7	7/7	3/3	7/9 GI 4/4 GII	4/4	6/6 GII 4/4 SaV	39/42	53/58
2015–16	3/3	3/3	20/23	23/26	4/4	4/4	1/1	2/3 GI 4/4 GII	3/3	4/5 GII 2/3 SaV	31/34	41/48
Total	9/15	9/15	161/202	164/205	19/24	20/24	4/4	17/20	7/7	16/18	201/252	225/282

**Table A2:** Recombination analysis of NoV and GenBank reference strains. Recombination events were identified using RDP4.

NoV Genotype	Nb of Strains ^1^	Major Parent	Minor Parent	Breakpoint nt ^2^	Breakpoint Region	Methods Detected ^4^	*p*-Value(B)
GII.2[P16]	1	MK762637	DQ456824	5096	Junction ^3^	R, G, B, M, C, T	1.245 × 10^−26^
GII.4 Syd[P4 NO]	18	GU445325.2	JX459908	4976	ORF1	G, B, M, C, S, T	6.105 × 10^−10^
GII.4 Syd[P4 NO]	1	GU445325.2	JX459908	4961	ORF1	G, B, M, C, T	2.286 × 10^−11^
GII.1[P33]	2	GQ845370.2	U07611.2	5064	ORF1	R, G, B, M, C, S, T	1.226 × 10^−9^
GII.4 Syd[P16]	7	AY772730	JX459908	5095	Junction ^3^	R, G, B, M, C, S, T	8.942 × 10^−29^
GII.6[P7]	4	MH218692	AB039778	5028	Junction ^3^	G, B, M, C, S, T	-
GII.13[P16]	2	MH218651	MK762637	5094	Junction ^3^	B, M, C, S, T	1.489 × 10^−31^
GII.4Syd[P31]	62	KT589391.1	MK762637	5080	Junction ^3^	R, G, B, M, C, S, T	1.986 × 10^−27^

^1^ Number of strains with evidence of same recombination event; ^2^ Position in major parent (Syd, Sydney 2012; NO, New Orleans 2009; SF, San Francisco); ^3^ ORF1/ORF2 junction; ^4^ Recombination detection methods (R, RDP; G, GENECONV; B, BootScan; M, MaxChi; C, Chimera; S, SiScan; T, 3 Seq).
