# Supplementary material for: Genetic Diversity of Norovirus and Sapovirus Outbreaks in Long-Term Care Facilities in Quebec, Canada, 2011–2016
Source: Viruses. 2026 Jan 8;18(1):85. doi: 10.3390/v18010085 (PMC12846708; doi:10.3390/v18010085)
Supplement: Supplementary file 1 [file viruses-18-00085-s001.zip › viruses-4043799-supplementary.pdf]

Table S1. NCBI Reference Genomes Used for NoV and SaV Primer Design

Assay	Target Gene	NCBI Accession Number
NoV GI	RdRp	L07418
NoV GI	RdRp	FJ515294
NoV GI	RdRp	JQ388274
NoV GI	RdRp	AB081723
NoV GI	RdRp	AF093797
NoV GI	RdRp	L07418
NoV GI	RdRp	JX023285
NoV GI	RdRp	NC_001959
NoV GI	RdRp	M87661
NoV GI	RdRp	L23828
NoV GI	RdRp	AB042808
NoV GI	RdRp	AB039774
NoV GI	RdRp	AB187514
NoV GI	RdRp	JX846929
NoV GI	RdRp	JN603244
NoV GI	RdRp	KM289169
NoV GI	RdRp	EF529738
NoV GI	RdRp	U04469
NoV GI	RdRp	GU299761
NoV GI	RdRp	AB290150
NoV GI	RdRp	HQ637267
NoV GI	RdRp	JN603254
NoV GI	VP1	L07418
NoV GI	VP1	FJ515294
NoV GI	VP1	JQ388274
NoV GI	VP1	AB081723
NoV GI	VP1	AF093797
NoV GI	VP1	AJ277610
NoV GI	VP1	JX023285
NoV GI	VP1	NC_001959
NoV GI	VP1	M87661
NoV GI	VP1	L23828
NoV GI	VP1	AJ313030
NoV GI	VP1	AB042808
NoV GI	VP1	JN603245
NoV GI	VP1	AB022679
NoV GI	VP1	AJ277621
NoV GI	VP1	AB039774
NoV GI	VP1	AJ277616
NoV GI	VP1	AJ277615
NoV GI	VP1	AB187514

NoV GI	VP1	AB187514
NoV GI	VP1	JN603244
NoV GI	VP1	GQ856470
NoV GI	VP1	KM289169
NoV GI	VP1	GQ856471
NoV GI	VP1	GQ856471
NoV GI	VP1	EF547396
NoV GI	VP1	AJ277612
NoV GI	VP1	AY038598
NoV GI	VP1	U04469
NoV GI	VP1	U04469
NoV GI	VP1	GQ856473
NoV GI	VP1	GQ856473
NoV GI	VP1	AF414406
NoV GI	VP1	AM263418
NoV GI	VP1	AJ277614
NoV GI	VP1	AF538679
NoV GI	VP1	GU299761
NoV GI	VP1	GU296356
NoV GI	VP1	JN183159
NoV GI	VP1	HQ637267
NoV GI	VP1	AY675555
NoV GI	VP1	AJ844469
NoV GI	VP1	AJ277609
NoV GII	RdRp	DQ078814
NoV GII	RdRp	JX445153
NoV GII	RdRp	EF187497
NoV GII	RdRp	JX445157
NoV GII	RdRp	AB541322
NoV GII	RdRp	HQ009513
NoV GII	RdRp	GQ845367
NoV GII	RdRp	GU445325
NoV GII	RdRp	JQ613573
NoV GII	RdRp	JN595867
NoV GII	RdRp	AY587985
NoV GII	RdRp	AY502023
NoV GII	RdRp	AY485642
NoV GII	RdRp	EU310927
NoV GII	RdRp	JX445159
NoV GII	RdRp	EF684915
NoV GII	RdRp	AY032605
NoV GII	RdRp	AF145896
NoV GII	RdRp	X86557
NoV GII	RdRp	GQ845368
NoV GII	RdRp	AB220921
NoV GII	RdRp	AB220921

NoV GII	RdRp	DQ369797
NoV GII	RdRp	AB220922
NoV GII	RdRp	AB220923
NoV GII	RdRp	AB541325
NoV GII	RdRp	AB541319
NoV GII	RdRp	JX459908
NoV GII	RdRp	JX459907
NoV GII	RdRp	X76716
NoV GII	RdRp	AY038600
NoV GII	RdRp	EF126966
NoV GII	RdRp	EF126961
NoV GII	RdRp	AB294779
NoV GII	RdRp	AB220922
NoV GII	RdRp	AF504671
NoV GII	RdRp	AF504650
NoV GII	RdRp	HQ449728
NoV GII	RdRp	JQ613569
NoV GII	RdRp	AB354294
NoV GII	RdRp	AY237415
NoV GII	RdRp	EU794907
NoV GII	RdRp	GU991355
NoV GII	RdRp	GU980585
NoV GII	RdRp	EU187437
NoV GII	RdRp	U22498
NoV GII	RdRp	U02030
NoV GII	RdRp	LC037415
NoV GII	RdRp	KR083017
NoV GII	RdRp	AB983218
NoV GII	RdRp	KJ156329
NoV GII	RdRp	KJ196286
NoV GII	RdRp	KC597139
NoV GII	RdRp	GQ856476
NoV GII	RdRp	AY772730
NoV GII	RdRp	AY682551
NoV GII	RdRp	AY682552
NoV GII	RdRp	KC576911
NoV GII	RdRp	U07611
NoV GII	RdRp	AB190457
NoV GII	RdRp	AF190817
NoV GII	RdRp	AF397156
NoV GII	RdRp	KJ196288
NoV GII	RdRp	AY134748
NoV GII	RdRp	DQ456824
NoV GII	RdRp	X81879
NoV GII	RdRp	JX846925
NoV GII	RdRp	AB083780

NoV GII	RdRp	GQ856469
NoV GII	RdRp	AB233471
NoV GII	RdRp	AB212306
NoV GII	RdRp	JF697282
NoV GII	RdRp	KJ196291
NoV GII	RdRp	AB039778
NoV GII	RdRp	JX989075
NoV GII	RdRp	AF414409
NoV GII	RdRp	AB039777
NoV GII	RdRp	EU424333
NoV GII	RdRp	AB542917
NoV GII	RdRp	EU275779
NoV GII	RdRp	AY038599
NoV GII	RdRp	DQ379715
NoV GII	RdRp	DQ372863
NoV GII	RdRp	AB039780
NoV GII	RdRp	JX846926
NoV GII	RdRp	GQ856465
NoV GII	RdRp	EU921354
NoV GII	RdRp	EF529741
NoV GII	RdRp	DQ379714
NoV GII	RdRp	AY823304
NoV GII	RdRp	AY823305
NoV GII	RdRp	HM635128
NoV GII	RdRp	AB542915
NoV GII	RdRp	AY682549
NoV GII	RdRp	AY919139
NoV GII	RdRp	AB074893
NoV GII	RdRp	AB126320
NoV GII	RdRp	AY823306
NoV GII	RdRp	AY823306
NoV GII	RdRp	AY823307
NoV GII	RdRp	AY682550
NoV GII	RdRp	AB089882
NoV GII	RdRp	GQ856474
NoV GII	RdRp	AB360387
NoV GII	RdRp	AF315813
NoV GII	VP1	DQ078814
NoV GII	VP1	JX445153
NoV GII	VP1	EF187497
NoV GII	VP1	JX445157
NoV GII	VP1	AB541322
NoV GII	VP1	HQ009513
NoV GII	VP1	GQ845367
NoV GII	VP1	GU445325
NoV GII	VP1	JQ613573

NoV GII	VP1	JN595867
NoV GII	VP1	AY587985
NoV GII	VP1	AY502023
NoV GII	VP1	AY485642
NoV GII	VP1	EU310927
NoV GII	VP1	JX445159
NoV GII	VP1	EF684915
NoV GII	VP1	AY032605
NoV GII	VP1	AF145896
NoV GII	VP1	X86557
NoV GII	VP1	GQ845368
NoV GII	VP1	AB220921
NoV GII	VP1	AB220921
NoV GII	VP1	DQ369797
NoV GII	VP1	AB220922
NoV GII	VP1	AB220923
NoV GII	VP1	AB541325
NoV GII	VP1	AB541319
NoV GII	VP1	JX459908
NoV GII	VP1	JX459907
NoV GII	VP1	X76716
NoV GII	VP1	AY038600
NoV GII	VP1	EF126966
NoV GII	VP1	EF126961
NoV GII	VP1	EU876882
NoV GII	VP1	AB294779
NoV GII	VP1	DQ078794
NoV GII	VP1	GQ246792
NoV GII	VP1	AB303923
NoV GII	VP1	AB303929
NoV GII	VP1	KF060124
NoV GII	VP1	AB220922
NoV GII	VP1	AF504671
NoV GII	VP1	AF504650
NoV GII	VP1	HQ449728
NoV GII	VP1	JQ613569
NoV GII	VP1	AJ277618
NoV GII	VP1	AB032758
NoV GII	VP1	AF427118
NoV GII	VP1	AY237415
NoV GII	VP1	EU794907
NoV GII	VP1	GU991355
NoV GII	VP1	GU980585
NoV GII	VP1	EU187437
NoV GII	VP1	JN899244
NoV GII	VP1	U22498

NoV GII	VP1	U02030
NoV GII	VP1	LC037415
NoV GII	VP1	KR083017
NoV GII	VP1	AB983218
NoV GII	VP1	KJ156329
NoV GII	VP1	KJ196286
NoV GII	VP1	KC597139
NoV GII	VP1	AY502009
NoV GII	VP1	DQ438972
NoV GII	VP1	GQ856476
NoV GII	VP1	AY772730
NoV GII	VP1	AY502010
NoV GII	VP1	U07611
NoV GII	VP1	AJ277606
NoV GII	VP1	AB190457
NoV GII	VP1	AF190817
NoV GII	VP1	AF397156
NoV GII	VP1	KJ196288
NoV GII	VP1	AJ277607
NoV GII	VP1	AY134748
NoV GII	VP1	DQ456824
NoV GII	VP1	X81879
NoV GII	VP1	JX846925
NoV GII	VP1	AB083780
NoV GII	VP1	GQ856469
NoV GII	VP1	AB212306
NoV GII	VP1	AJ844470
NoV GII	VP1	KJ196291
NoV GII	VP1	AB039778
NoV GII	VP1	JX989075
NoV GII	VP1	AJ277620
NoV GII	VP1	AJ277608
NoV GII	VP1	AF414409
NoV GII	VP1	AB039777
NoV GII	VP1	EU373815
NoV GII	VP1	EU424333
NoV GII	VP1	AB542917
NoV GII	VP1	AY038599
NoV GII	VP1	DQ379715
NoV GII	VP1	AB039780
NoV GII	VP1	AF195848
NoV GII	VP1	AY130761
NoV GII	VP1	GQ856465
NoV GII	VP1	AY113106
NoV GII	VP1	AB078334
NoV GII	VP1	EU921354

NoV GII	VP1	DQ379714
NoV GII	VP1	AY823304
NoV GII	VP1	AY823305
NoV GII	VP1	AY675554
NoV GII	VP1	AB542915
NoV GII	VP1	AB074893
NoV GII	VP1	AB126320
NoV GII	VP1	AY823306
NoV GII	VP1	AY823306
NoV GII	VP1	AY823307
NoV GII	VP1	AY130762
NoV GII	VP1	GQ856474
SaV	RdRp	AY237420
SaV	RdRp	AY603425
SaV	RdRp	AB455793
SaV	RdRp	AY646855
SaV	RdRp	AJ249939
SaV	RdRp	AY237419
SaV	RdRp	U95645
SaV	RdRp	DQ058829
SaV	RdRp	DQ366346
SaV	RdRp	AF435814
SaV	RdRp	X86560
SaV	RdRp	AY237422
SaV	RdRp	AY694184
SaV	RdRp	U65427
SaV	RdRp	DQ366345
SaV	RdRp	AB614356
SaV	RdRp	FJ844411
SaV	RdRp	U73124
SaV	RdRp	AY646856
SaV	RdRp	DQ366344
SaV	RdRp	AB924385
SaV	RdRp	AB775659
SaV	VP1	AY237420
SaV	VP1	AY603425
SaV	VP1	AB455793
SaV	VP1	AY646855
SaV	VP1	AJ249939
SaV	VP1	AY237419
SaV	VP1	U95645
SaV	VP1	DQ104360
SaV	VP1	AF435812
SaV	VP1	AB429084
SaV	VP1	AY289804
SaV	VP1	AB630067

SaV	VP1	DQ058829
SaV	VP1	DQ366346
SaV	VP1	AF435814
SaV	VP1	AB455795
SaV	VP1	AB448753
SaV	VP1	AB448766
SaV	VP1	DQ104357
SaV	VP1	X86560
SaV	VP1	AY237422
SaV	VP1	AY694184
SaV	VP1	U65427
SaV	VP1	DQ366345
SaV	VP1	AB253740
SaV	VP1	AB614356
SaV	VP1	FJ844411
SaV	VP1	AB607855
SaV	VP1	U73124
SaV	VP1	AJ606693
SaV	VP1	AJ606694
SaV	VP1	AB455803
SaV	VP1	AJ606696
SaV	VP1	AF435813
SaV	VP1	AB258428
SaV	VP1	AB522390
SaV	VP1	AY646856
SaV	VP1	DQ366344
SaV	VP1	AB924385
SaV	VP1	AY289803
SaV	VP1	AB775659
